# The Influence of Age and Gender on Skin-Associated Microbial Communities in Urban and Rural Human Populations

**DOI:** 10.1371/journal.pone.0141842

**Published:** 2015-10-28

**Authors:** Shi Ying, Dan-Ning Zeng, Liang Chi, Yuan Tan, Carlos Galzote, Cesar Cardona, Simon Lax, Jack Gilbert, Zhe-Xue Quan

**Affiliations:** 1 Department of Microbiology and Microbial Engineering, School of Life Science, Fudan University, Shanghai, China; 2 Scientific Affairs, Johnson & Johnson China Ltd., Shanghai, China; 3 Graduate Program in Biophysical Sciences, University of Chicago, Chicago, Illinois, United States of America; 4 Department of Ecology and Evolution, University of Chicago, Chicago, Illinois, United States of America; 5 Institute for Genomic and Systems Biology, Argonne National Laboratory, Argonne, Illinois, United States of America; 6 Marine Biological Laboratory, Woods Hole, Massachusetts, United States of America; 7 College of Environmental and Resource Sciences, Zhejiang University, Hangzhou, China; National Cancer Institute, UNITED STATES

## Abstract

Differences in the bacterial community structure associated with 7 skin sites in 71 healthy people over five days showed significant correlations with age, gender, physical skin parameters, and whether participants lived in urban or rural locations in the same city. While body site explained the majority of the variance in bacterial community structure, the composition of the skin-associated bacterial communities were predominantly influenced by whether the participants were living in an urban or rural environment, with a significantly greater relative abundance of *Trabulsiella* in urban populations. Adults maintained greater overall microbial diversity than adolescents or the elderly, while the intragroup variation among the elderly and rural populations was significantly greater. Skin-associated bacterial community structure and composition could predict whether a sample came from an urban or a rural resident ~5x greater than random.

## Introduction

As the largest organ of the human body, skin is a highly variable microbial habitat colonized by a broad diversity of bacteria and fungi [1]. These assemblages demonstrate significant intra- and inter-individual variation [2, 3] and topographical and temporal diversity [4, 5]. Additionally, gender [6] and cohabitation with other humans or animals [7] have been shown to shape the skin microbiome. The skin is our primary interface with the physical world, and as such the variability in skin microbial communities within a human population may be influenced by place of residence, as well as skin type (sebaceous, dry and moist), age, and gender, though the extent to which these factors influence this variability remains unknown.

The place of residence of a human population is associated with the composition of human-associated microbial communities, for example populations living in Venezuela and the United States have significantly different skin and stool-associated microbiomes [8]. However, these populations likely have different lifestyles and diets that will influence the structure of their microbial assemblages, making it difficult to disentangle the specific factors influencing the structure and composition of their microbiota. Even in the same country, due to the different diets and lifestyles, gut-associated microbiomes have been observed to have different composition and structure between urban and rural populations [9]. Recent evidence suggests that when an individual changes their city of residence for more than a month (Boston, USA to Bangkok, Thailand), it can have a significant impact on the structure and composition of their saliva and stool microbiota [10]. However, skin-associated microbiome, thought of as highly variable, does not seem to be influenced by long-term changes in immediate habitat for human populations. For example, when families move between different houses, there is no observable shift in their skin-associated microbial composition [11]. Here we explore whether the skin microbiome of a human population within a single city demonstrates biogeographic differentiation.

Cutaneous bacterial communities associated with 7 skin sites from 71 healthy individuals living in rural and urban areas of Shanghai, China, were examined in the context of several population variables. Correlations between bacterial community structure and skin physical parameters such as sebum, trans-epidermal water loss (TEWL), moisture and pH were also examined. Using these data, we tested the hypothesis that within a geographically semi-contiguous human population, the skin microbiota can show regional biogeographic patterns.

## Materials and Methods

### Ethics statement

This study was approved by the Ethical Committee of Fudan University prior to implementation. A written informed consent was obtained from each subject or their guardians prior to sample collection. All data were de-identified.

### Sampling

A total of 71 subjects were recruited for the study, 36 living in the urban regions and 35 living in the rural regions of Shanghai, China (Table 1). The rural adults and elderly included in this study were all agricultural field-workers, whereas most urban participants had indoor occupations. Moreover, no subjects lived in the same family or worked in the same office. All subjects were classified into 3 age groups: elderly (50~60 years old), adults (25~35 years old) and adolescents (12~19 years old). Medical and medication history were obtained for each individual by questionnaires; additionally, a complete dermatologic examination was performed. Subjects with any history of dermatologic diseases and those who had any antibiotic exposure in the past 6 months were excluded. Each subject was instructed not to wash the specific body sites for 12 hours (except hands for 2 hours) prior to sampling. Seven skin sites were sampled on each subject in order: back of hands (Hb), interdigital web space (Is), volar forearm (Vf), antecubital fossa (Af), nares (Na), glabella (Gb) and back (Ba). For symmetrical sites, the selections of sampling were random. Samples were collected in a temperature and humidity controlled rooms. Sample collection was performed in August of 2011. A 4-cm^2^ area (for Is an ~1-cm^2^ area) was swabbed with polyester fiber-headed swabs moistened with solution of 0.15 M NaCl and 0.1% Tween 20 [6, 12]. The sampling regions were swabbed approximately 50 times for at least 30 seconds. Then the swab head was picked off by sterilized tweezers and carefully placed in the PowerBead Tube of the MO BIO PowerSoil DNA Isolation Kit (MO BIO Lab, Carlsbad, CA, USA). The nares were sampled with a twisting motion, gently rubbing the mucosal surfaces of the anterior nares with a sterile and moistened swab, covering the area twice. All samples were stored at 4°C for DNA extraction. Following skin sampling, measurements were taken of skin sebum (by Sebumeter^®^ SM 810, Courage & Khazaka, Cologne, Germany), TEWL (by VapoMeter, Delfin Tech, Kuopio, Finland), moisture (by Corneometer^®^ CM 825, Courage & Khazaka) and pH (by Skin-pH-Meter^®^ PH 905, Courage & Khazaka). An unused moistened swab head (negative control) was placed in another PowerBead Tube. Three replicate swabs of the identified body sites were taken from each subject with a 1-day interval (Monday-Wednesday-Friday) between each sampling.

**Table 1:** Subject information.

Age	Residence	Gender	No. of subjects	Average-age
Elderly	Urban	Male	5	54.4
		Female	6	53.0
	Rural	Male	5	53.2
		Female	6	53.5
Adults	Urban	Male	6	29.0
		Female	6	31.0
	Rural	Male	6	30.2
		Female	6	31.3
Adolescents	Urban	Male	6	14.3
		Female	7	15.9
	Rural	Male	6	16.0
		Female	6	17.0

### DNA extraction

DNA extraction from the head of the swabs was performed within 12 hours of sampling. The MO BIO PowerSoil DNA Isolation Kit with modifications was applied [4]. To each PowerBead tube, 60 μl of solution C1 were added, the tube sealed, and it was then placed in a water bath at 65°C for 10 minutes. The tubes were then shaken horizontally for 2 minutes at maximum speed using the MO BIO Vortex Adapter. The remaining steps were performed as directed by the manufacturer. Extracted DNA was resuspended in 100 μl eluent and stored at −20°C prior to PCR amplification.

### PCR amplification

Bacterial 16S rRNA genes were amplified from the extracted DNA using two stages of PCR. For the first round of PCR, the modified primer set 27FYM (5’-AGAGTTTGAT(C/T)(A/C)TGGCTCAG-3’) and 536RK (5’-GTATTACCGCGGC(G/T)GCTGG-3’) were applied. For each 25-μl reaction, PCRs consisted of 0.25 μl of each forward and reverse primer (10 μM), 6 μl of template DNA, 1 μl of bovine serum albumin (BSA, 20 mg/ml) and 12.5 μl of Ex Taq Premix version 2.0 (TaKaRa Biotech., Dalian, China). The samples were initially denatured at 94°C for 5 min, then amplified using 20 cycles of 94°C for 45 s, 53°C for 30 s, and 72°C for 90 s. A final extension of 10 min at 72°C was added at the end of the program. For the second round of PCR, the primer set, AdaA-27FYM and AdaB-536RK, which contained 454 pyrosequencing adapters were applied. The forward primer AdaA-27FYM (5’-CCATCTCATCCCTGCGTGTCTCCGACGACTNNNNNNNNTCAGAGTTTGAT(C/T)(A/C)TGGCTCAG-3’) contained 454 pyrosequencing adapter A, a unique 8-bp barcode (designated by NNNNNNNN) used to tag each PCR product [13], the bacterial primer 27FYM, and a 2-bp linker “TC” inserted between the barcode and the primer. The reverse primer AdaB-536RK (5’-CCTATCCCCTGTGTGCCTTGGCAGTCGACTCAGTATTACCGCGGC(G/T)GCTGG-3’) contained 454-pyrosequencing adapter B, the bacterial primer 536RK, and a “CA” inserted as a linker. For each 100-μl reaction, PCRs consisted of 1 μl of each forward and reverse primer (10 μM), 10 μl of template using the PCR product of the first round, 4 μl of BSA and 50 μl of Ex Taq Premix (TaKaRa). The second-round PCR program was similar to the first round, except that the number of amplification cycle was 10 instead of 20.

### PCR product purification and sample pooling

The PCR products of the second round were purified with UltraClean PCR CleanUp Kit (MO BIO Lab) following the direction of the manufacturer. The PCR products were finally re-suspended in 50 μl eluent and stored at −20°C. Amplicon DNA concentrations were measured using PicoGreen dsDNA reagent (Invitrogen, Grand Island, NY, USA) on a TBS-380 Mini-Fluorometer (Promega, Madison, WI, USA). Based on the quantification result, cleaned PCR amplicons that belong to the same pyrosequencing plate were added in equimolar ratios into a 1.5-ml tube. The composite sample was cleaned again using AxyPrep DNA Gel extraction Kit (Axygen, Tewksbury, MA, USA). The purified PCR products were sequenced using a GS-FLX pyrosequencing platform with Titanium chemistry (Roche, Basel, Switzerland) following the direction of the manufacturer.

### Sequence analysis

Sequences were processed using the QIIME (http://www.qiime.org) software package [14]. Reads were assigned to particular libraries according to the 8-nucletide (nt) barcodes with the criteria of higher than 25 quality value, >250 nt in length, no ambiguous characters and no homopolymers run exceeding 8 nt. The complete data set was chimera-checked using USEARCH61 (http://drive5.com/usearch/usearch_docs.html) with the Greengenes database [15]. Then the remaining reads were clustered into operational taxonomic units (OTUs) by UCLUST [16] based on 97% identity. After singletons removal, a representative sequence was chosen from each OTU by selecting the first sequence (the UCLUST cluster seed). Taxonomy was assigned to each representative sequence using the Ribosomal Database Project (RDP) classifier [17], with a minimum confidence of 80%. Representative sequences were aligned against the Greengenes database using Python Nearest Alignment Space Termination tool (PyNAST) [18], and used a minimum alignment length of 210 and a minimum identity of 75%. The OTUs which failed to align to representative sequences were dropped. The PH Lane mask was used to remove hypervariable regions after alignment. The aligned representative sequences were assigned a phylogenetic relationship using FastTree [19]. To ensure adequate representation of the community structure, samples with <200 reads were removed. To evaluate the amount of diversity contained within communities (alpha diversity), rarefaction analysis was performed with Chao1, Shannon and phylogenetic distance (PD) index [20]. To determine the amount of diversity shared between two communities (beta diversity), UniFrac distances [21] were calculated between all pairs of samples. UniFrac distances were based on the fraction of branch length shared between two communities in a phylogenetic tree. Unweighted UniFrac accounts for membership only (community membership, not considering the content of each member), whereas weighted UniFrac accounts for membership and relative abundance (community structure, considering members and the content of each member together). UniFrac-based jackknifed hierarchical clustering was performed using unweighted pair group method with arithmetic mean (UPGMA) in QIIME. Principal coordinates analysis (PCoA) was also performed on the UniFrac distance matrices, and visualized using the KiNG graphics program (http://kinemage.biochem.duke.edu/software/king.php). We subsampled 1,364 samples to 200 sequences per sample, and then collapsed rarefied samples into 84 groups according to factors of age, gender, residence, and skin site. Again, we rarefied the 84 groups to 1,400 sequences per group. Finally, these rarefied groups were used to perform PCoA and UPGMA analysis; the relative abundances of these groups were all examined using heat maps. Except for these analyses, all other investigations used all 1,364 of the rarefied samples. The sequence data generated for this study were deposited in the NCBI GenBank Short Read Archive (SRA) under accession number SRP051059.

### Statistical analysis

We performed *t*-test on alpha diversity and UniFrac distance of different categories, Analysis of Variance (ANOVA) on OTU abundance of different categories, Pearson correlation on environmental factors and genus-level abundances, Mantel test on correlation of skin physical parameters and UniFrac distance matrices, and Analysis of Similarities (ANOSIM) on UniFrac distance matrices of different categories. All these statistical methods were performed in QIIME. All *P* values of ANOVA and Pearson correlation were corrected used the Bonferroni method for multiple comparisons. We used random forest supervised learning models to determine the extent to which skin-associated microbial communities could be used to predict the age, gender, place of residence, or skin surface environment of the subject from whom a sample was taken. These models formed decision trees using a subset of samples to identify patterns associated with a metadata category, and then the accuracy of the tree was tested on the remaining samples not used for training. Each model ran 1000 independent trees and reports the ratio of model error to random error as a metric for the predictive power of the category’s microbial communities. A greater ratio of baseline-to-model error indicates a better ability to classify that grouping by microbial community alone. Triplicate samples were pooled and rarified to an even depth of 400 reads, resulting in a total of 479 samples. OTUs detected in less than 10 samples were discarded. All models were run with 10-fold cross-validation using the *supervised_learning*.*py* script in QIIME.

## Results

**Fig 1:**
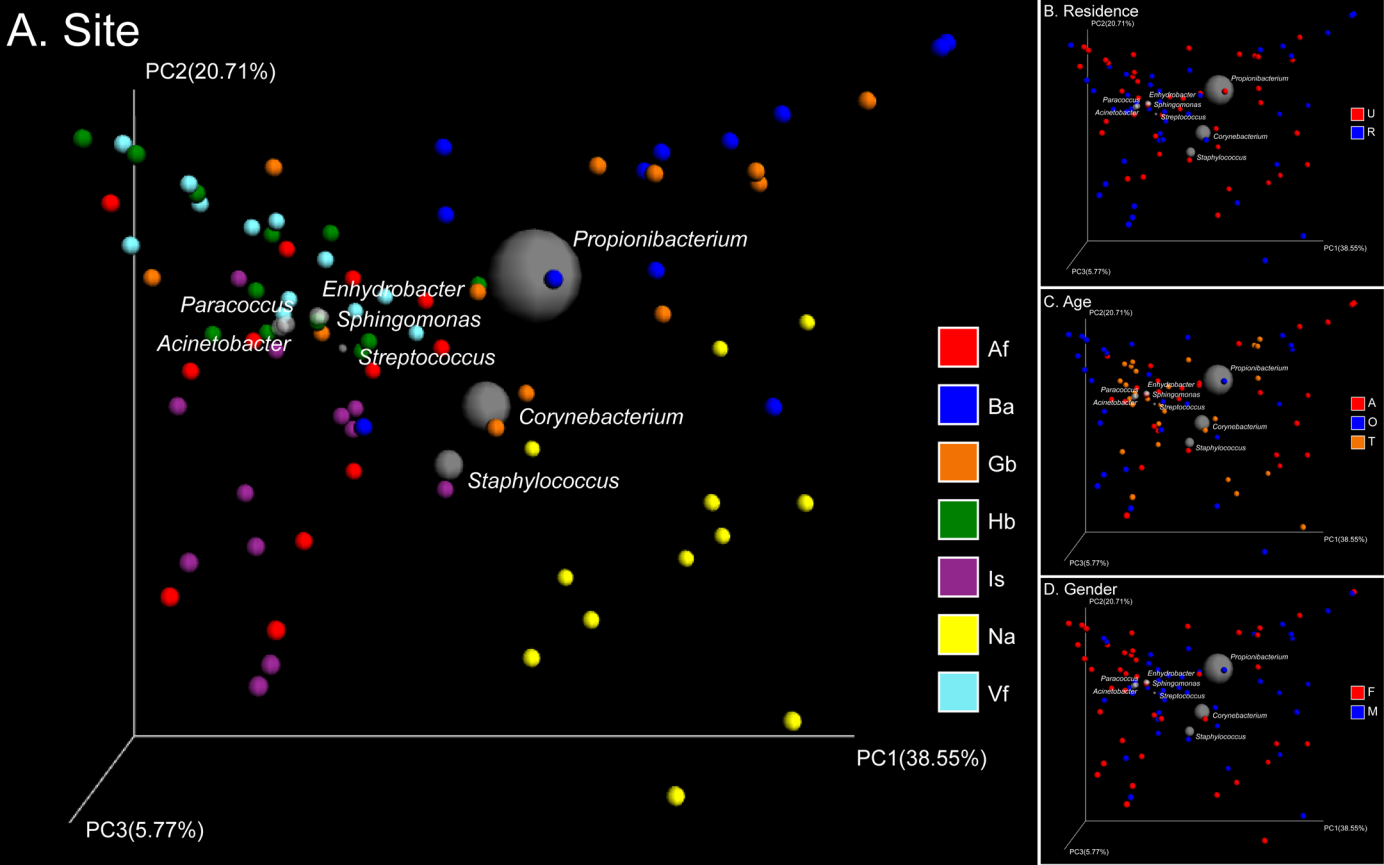
PCoA analysis of 84 pooled groups based on weighted UniFrac distances. Clustering of study subjects using principal coordinates analysis (PCoA) based on weighted UniFrac distances. In the Bi-plot, 8 predominant genera are indicated by the size of the gray circle representing the abundance of the taxon. Each point corresponds to a group colored by (A) site, (B) residence, (C) age, or (D) gender. In the abbreviation of group names, A: adult, T: adolescent, O: elderly; F: female, M: male; U: urban populations, R: rural populations; Hb: back of hands, Is: interdigital web space, Vf: volar forearm, Af: antecubital fossa, Na: nares, Gb: glabella, Ba: back.

**Fig 2:**
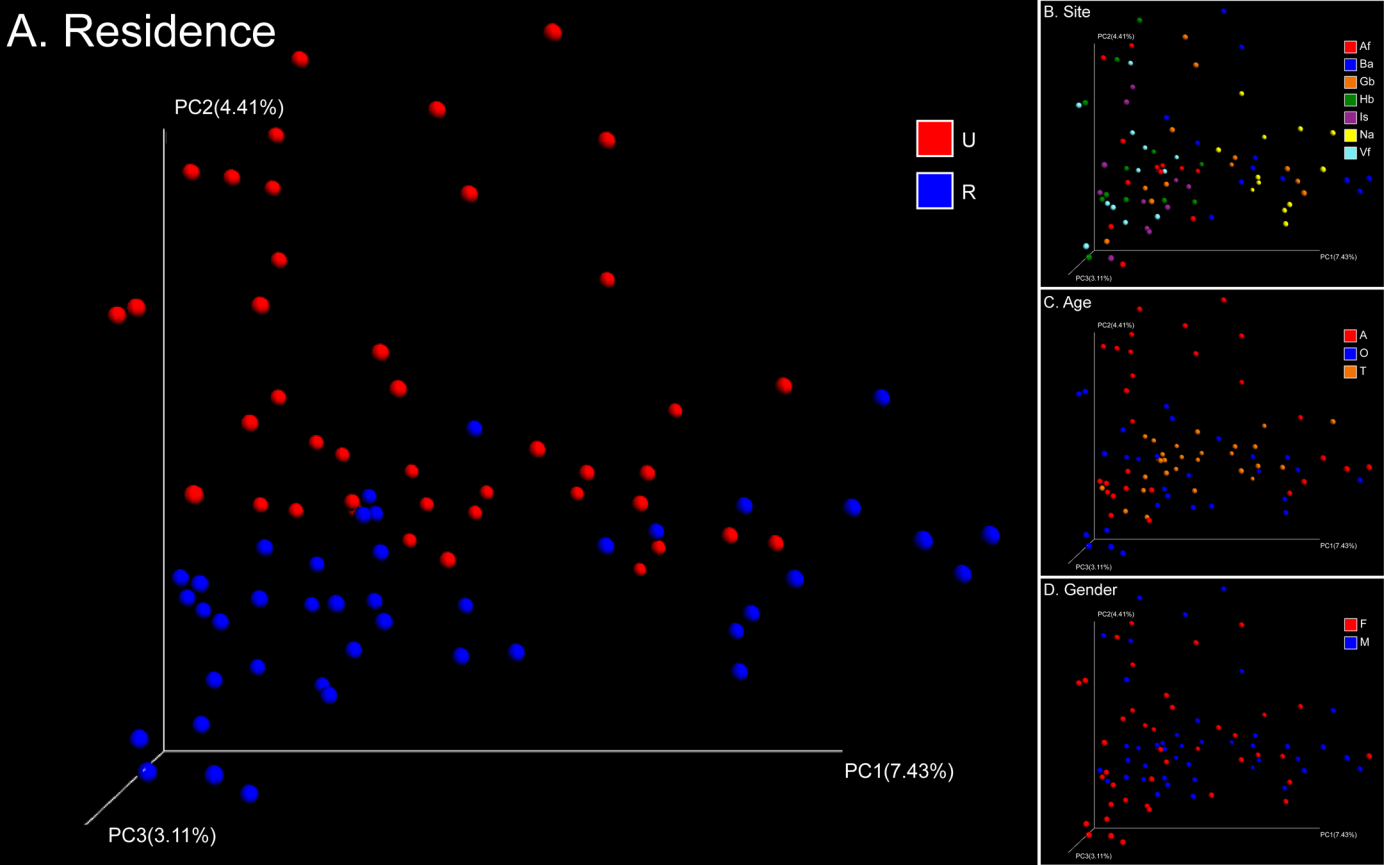
PCoA analysis of 84 pooled groups based on unweighted UniFrac distances. Clustering of study subjects using principal coordinates analysis (PCoA) based on unweighted UniFrac distances. Each point corresponds to a group colored by (A) residence, (B) site, (C) age, or (D) gender. The abbreviations and the corresponding explanations are given in Fig 1.

Skin samples from 71 participants (Table 1) generated a total of 625,372 high-quality 16S rRNA gene sequences from 1,364 samples (mean 458 sequence reads per sample). These sequences clustered into 13,004 OTUs (S1 Fig). The main genera were *Propionibacterium*, *Corynebacterium*, *Staphylococcus*, *Streptococcus*, *Enhydrobacter*, *Sphingomonas*, *Paracoccus*, and *Acinetobacter*. Weighted and unweighted UniFrac distances showed clear differentiation of the bacterial community structure and membership between different subsets of the population studied (S2 Fig). The primary factor describing variation in the bacterial community structure (the relative abundance of different taxa) was skin site followed by the age of participants, then gender, and finally place of residence (ANOSIM *R* = 0.24, 0.05, 0.02, 0.02, all *P* = 0.001 with weighted UniFrac, Fig 1). Meanwhile the factors that correlated with changes in community membership (community composition) were place of residence and skin site followed by age and gender (ANOSIM *R* = 0.20, 0.18, 0.08, 0.02, all *P* = 0.001 with unweighted UniFrac, Fig 2). These factors could partially interpret differences between each sub-population (S1 Table).

**Fig 3:**
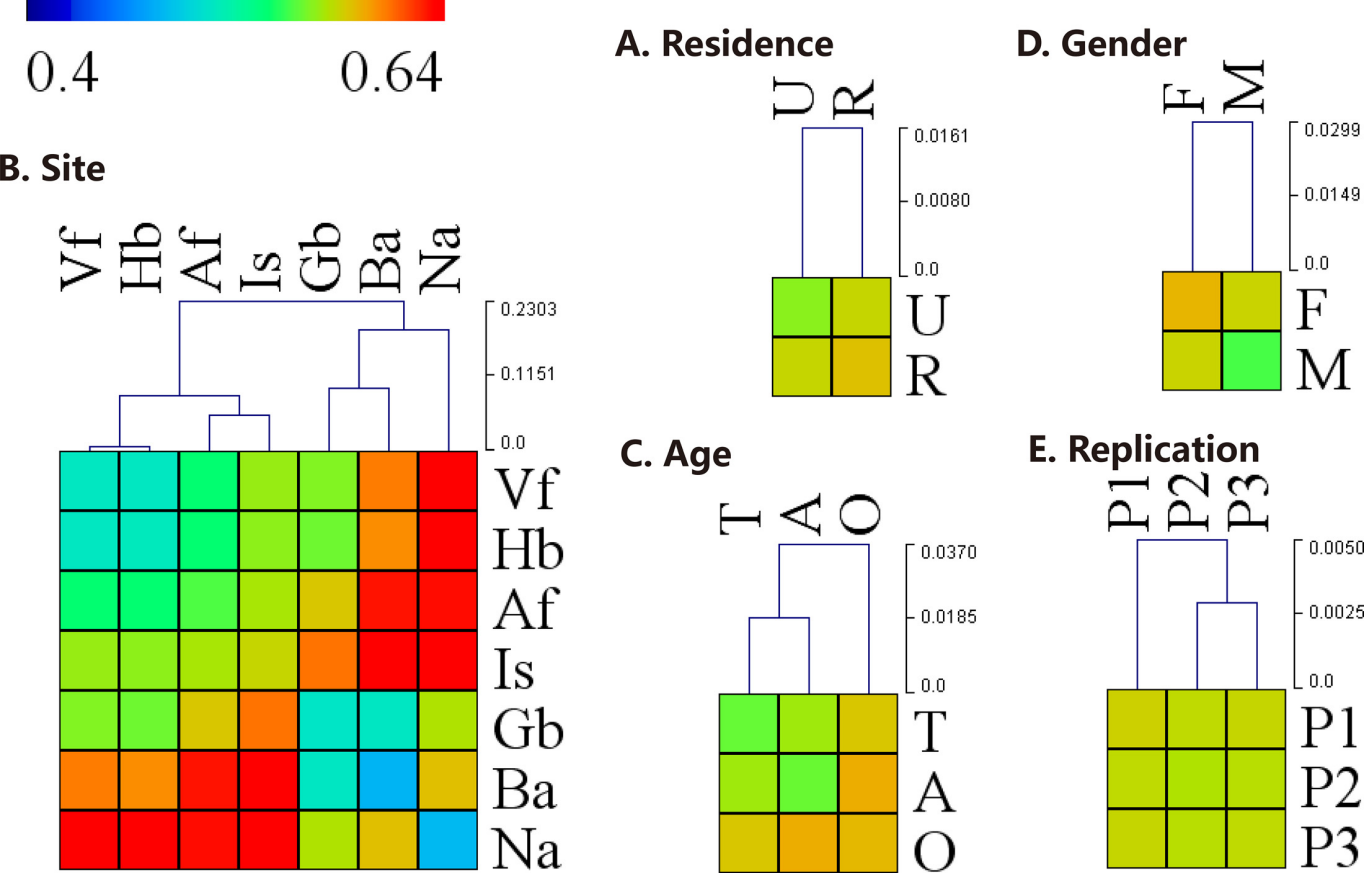
Hierarchical-clustering heat-map of the inter- and intra-groups distance. Hierarchical-clustering heat-map of the weighted UniFrac pairwise distance between several groups and the clustering dendrogram using Euclidean distance by (A) residence, (B) site, (C) age, (D) gender, or (E) replication. Blue and Red cells represents low and high distance values, respectively. The inter- and intra-groups distances revealed community differences between groups. The abbreviations and the corresponding explanations are given in Fig 1. P1, P2, P3: three replicate sampling.

**Fig 4:**
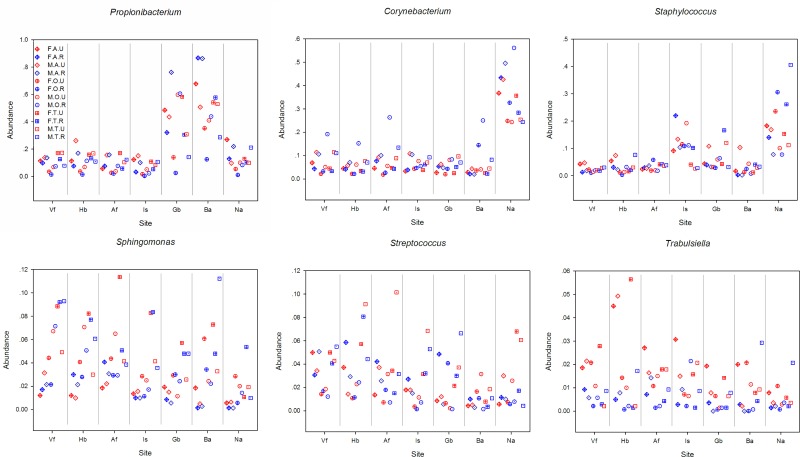
The relative abundance of different bacterial genera in different groups. All samples are combined to 12 groups (FAU, FAR, MAU, MAR, FOU, FOR, MOU, MOR, FTU, FTR, MTU, MTR) based on gender, age and residence. The abbreviations and the corresponding explanations are given in Fig 1. For example, FAU means the group involved samples that were from female (F) adults (A) living in an urban (U) area.

Urban and rural populations had similar skin-associated bacterial richness estimates (Table 2, S3 Fig). However, the intragroup variation in microbial community structure among rural subjects was significantly greater than urban subjects (two-tailed t-test, *P* < 10^−4^, Fig 3A). Unweighted UniFrac clearly showed the separation of different groups based on urban versus rural residency (Fig 2A). The relative abundance of *Trabulsiella* was significantly greater in urban dwellers compared to rural dwellers (1.5% on urban dwellers and 0.5% on rural dwellers, ANOVA *P* < 10^−4^), especially on sites including Hb, Vf and Gb (All the results of ANOVA are shown in S2 Table and the relative abundance of different bacterial genera in different group are shown in Fig 4). Among the adults, *Propionibacterium* on Is of urban dwellers (14.0%) was significantly greater than rural dwellers (6.5%, ANOVA *P* = 0.03). However, *Propionibacterium* on Ba was significantly more abundant in rural dwellers (57.2% on urban adults and 86.2% on rural adults, ANOVA *P* = 0.0008). For the females, the content of *Propionibacterium* on Gb of urban dwellers (42.5%) was significantly greater than that of rural dwellers (21.8%, ANOVA *P* = 0.02), whereas *Corynebacterium* showed an opposite pattern (2.5% and 5.1%, respectively, ANOVA *P* = 0.003).

**Table 2:** Analysis of alpha diversity.

	Chao1	Phylogenetic Distance (PD)	Shannon
**Residence**	U,R ^ns^ ^a^	U>R *	U,R ^ns^
**Site**	Vf>Gb,Ba,Na ** ^b^	Vf>Is,Gb,Ba,Na **	Vf>Is,Gb,Ba,Na **
	Hb>Gb,Ba,Na **	Hb>Is,Gb,Ba,Na **	Hb>Is,Gb,Ba,Na **
	Af>Gb,Ba,Na **	Af>Gb,Ba,Na **	Af>Gb,Ba,Na **
	Is>Gb,Ba,Na **	Is>Gb,Ba,Na **	Is>Gb,Ba,Na **
	Gb>Ba,Na **	Gb>Ba,Na **	Gb>Ba,Na **
**Age**	A>T,O **	A>O,T *	A,O,T ^ns^
**Gender**	F,M ^ns^	F,M ^ns^	F,M ^ns^
**Replication**	P1,P2,P3 ^ns^	P1,P2,P3 ^ns^	P1,P2,P3 ^ns^

^a^T-test results: ns: not significant,

*: 0.001< P<0.05,

**: P<0.001.

^b^Vf>Gb, Ba, Na means the diversity index value of Vf is significantly higher than Gb, Ba and Na respectively.

In the comparison of different sites, Table 2 just lists the significant results. The abbreviations and the corresponding explanations are given in Fig 1. P1, P2, P3: three replicate sampling.

**Fig 5:**
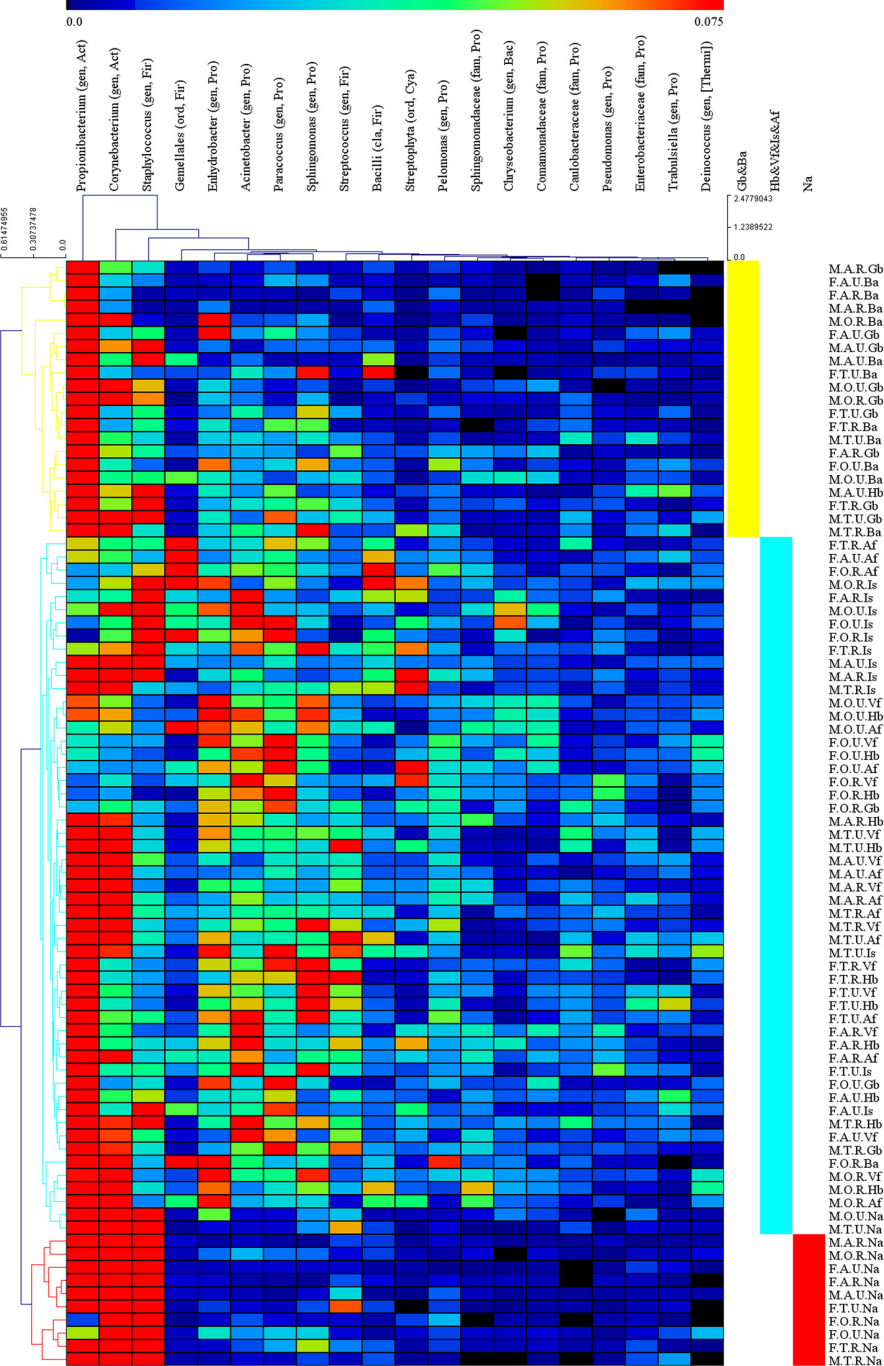
Hierarchical-clustering heat-map of the relative abundance of the 20 most abundant bacterial genera. Hierarchical-clustering heat-map of the relative abundance and the clustering dendrogram of different groups and the 20 most abundant bacterial genera, using Euclidean distance. Color intensity indicates abundance, ranging from black (absence), blue (low abundance) to red (high abundance). All of samples are combined to 84 groups based on age, gender, residence, and skin site. The abbreviations and the corresponding explanations are given in Fig 1. For example, M.A.R.Gb means the group involved samples which are from a rural (R) male (M) adult’s (A) glabella (Gb). Classifications are to the genus (gen), family (fam), order (ord) or class (cla) level. For each taxon, the phylum is also indicated: Act, Actinobacteria; Bac, Bacteroidetes; Cya, Cyanobacteria; Fir, Firmicutes; Pro, Proteobacteria. Taxa are classified to the highest taxonomic level to which they were confidently assigned. For the details see S3 Table.

Na and sebaceous sites (Gb and Ba) showed significantly lower alpha diversity than dry (Vf and Hb) and moist (Af and Is) sites (Table 2, S3 Fig). The 1,364 samples were clustered into 84 groups based on the 4 key experimental factors: age, gender, place of residence and skin site. Then we used hierarchical-clustering heat-map to analysis the 20 most abundant bacterial genera (those with >1% relative abundance) of the 84 pooled groups (Fig 5). Three main clusters were defined based on the sebaceous skin sites, the nares that clustered separately, whereas dry and moist sites clustered together (Fig 5). Clustering based on weighted UniFrac distances demonstrated grouping by Ba and Gb, Is and Af, Vf and Hb, and Na that clustered separately (Fig 1A, S2A Fig).

Nares-associated microbial communities comprised *Corynebacterium* (35.5%), *Staphylococcus* (17.7%), and *Alloiococcus* (5.2%), which were all significantly higher than in other skin sites (ANOVA all *P* < 10^−4^). Ba showed lowest intragroup variation, whereas Is demonstrated the greatest difference in community structure between subjects (Fig 3B). Interestingly, the two dry skin sites, Vf and Hb, demonstrated as much variation in microbial community structure within a single site as they did between sites (Fig 3B). Furthermore, there was no significant difference in the relative abundance of the dominant bacterial genera between sites Vf and Hb (ANOVA all *P* > 0.05). In the moist sites, *Staphylococcus* showed the greatest difference in relative abundance (3.2% on Af and 11.3% on Is, ANOVA *P* < 10^−4^), whereas *Propionibacterium* in the sebaceous sites showed the greatest difference (53.4% on Ba and 40.5% on Gb, ANOVA *P* < 10^−4^) and highest relative abundance across all sites.

Bacterial alpha diversity was significantly different between age groups (Table 2, S3 Fig). Adults maintained a greater overall diversity than adolescents and the elderly (all *P* < 10^−4^ with Chao1, S4 Table). For the male rural group, elderly individuals had the lowest alpha diversity, which was significantly different from adolescents and adults (*P* = 2.7 × 10^−3^, < 1.0 × 10^−4^ with Chao1, S4 Table). Among the different age groups, the intragroup variation was significantly greater among the elderly compared to adolescents and adults (all *P* < 10^−4^), whereas the bacterial population of adults was more similar to adolescents than elderly populations (Fig 3C). Ba showed the strongest correlation of bacterial community structure with age (ANOSIM *R* = 0.15, *P* = 0.001 with weighted UniFrac, S1 Table). Within the age groupings, *Enhydrobacter* was significantly more abundant among elderly individuals (5.8%) compared to adolescents (3.3%, ANOVA *P* < 10^−4^) and adults (2.4%, ANOVA *P* < 10^−4^), especially on sites Ba and Na. On the other hand, the relative abundance of *Propionibacterium* among the elderly (12.6%) was significantly lower than adolescents (21.9%, ANOVA *P* < 10^−4^) and adults (26.7%, ANOVA *P* < 10^−4^), especially on dry and moist sites. Meanwhile, on site Is, the relative abundance of *Sphingomonas* and *Streptococcus* was significantly greater among adolescents (6.3% and 4.6%) compared to adults (1.3%, ANOVA *P* < 10^−4^; and 1.8%, ANOVA *P* = 10^−4^) and elderly (2.1%, ANOVA *P* = 10^−4^; and 0.7%, ANOVA *P* < 10^−4^).

Bacterial community richness was similar between males and females (Table 2, S3 Fig). However, on site Gb, elderly males showed significantly lower bacterial alpha diversity compared with elderly females (*P* < 10^−4^ with Chao1, S4 Table). Additionally, rural males had a significantly lower alpha diversity than rural females (*P* = 6.7 × 10^−4^ with Chao1), especially on site Gb (S4 Table). Interestingly, the intragroup variation in microbial community structure was significantly greater among females than among males (*P* < 10^−4^, Fig 3D). The relative abundance of *Propionibacterium* was significantly greater in males compared to females (23.5% on male and 18.2% on female, ANOVA *P* = 0.01), especially on Gb (especially among elderly subjects) and Hb (especially among adults and elderly). On Gb of the elderly, the relative abundance of *Propionibacterium* was 7 times greater in males (60.2%) compared to females (9.3%, ANOVA *P* < 10^−4^). Additionally, the relative abundance of *Corynebacterium* in males was significantly greater than females (13.1% on male and 8.4% on female, ANOVA *P* < 10^−4^), especially on Hb, Vf, Af, Gb and Is. The relative abundance of *Acinetobacter*, *Paracoccus* (especially on Ba of elderly) and *Sphingomonas* in females (4.5% and 4.3%, respectively) was significantly greater than males (2.8%, ANOVA *P* < 10^−4^; and 2.8%, ANOVA *P* < 10^−4^). For the Na of elderly, the relative abundance of *Staphylococcus* and *Alloiococcus* was 3 to 7 times greater in females (26.9% and 15.4%, respectively) than males (9.3%, ANOVA *P* = 0.009; and 2.6%, ANOVA *P* = 0.02), whereas *Anaerococcus* was significantly greater in males than females (4.0% and 1.0%, respectively, ANOVA *P* = 0.004). Among adolescents, site Af maintained 3 times the relative abundance of *Streptococcus* in males (8.7%) compared to females (2.6%, ANOVA *P* = 0.03).

A Mantel test was performed to determine the possible correlations between microbial distributions on skin sites and skin physicochemical parameters (Table 3). Positive correlations were observed between sebum content and both the bacterial community structure and membership on the sebaceous site Gb; and pH and the bacterial community structure on Af and community membership on Vf, Af, Gb and Ba. Changes in pH were positively correlated with changes in the relative abundance of *Corynebacterium* (*R* = 0.29, *P* = 6.0 × 10^−20^; especially on Hb, Af, Ba and Vf; S5 Table), whereas sebum content (*R* = 0.36, *P* = 2.1 × 10^−20^, especially on Gb) and moisture (*R* = 0.38, *P* = 1.0 × 10^−35^) were positively correlated with the relative abundance of *Propionibacterium*. Finally, TEWL was significantly correlated with the relative abundance of *Staphylococcus* (*R* = 0.14, *P* = 1.3 × 10^−4^).

**Table 3:** Mantel-test of UniFrac matrix and skin physical parameters.

	Sebum				Moisture				Trans-epidermal water loss (TEWL)				pH			
	unweighted		weighted		unweighted		weighted		unweighted		weighted		unweighted		weighted	
	R	*P*	R	*P*	R	*P*	R	*P*	R	*P*	R	*P*	R	*P*	R	*P*
**All** ^a^	0.005	0.797	-0.019	0.332	0.025	0.084	0.077	0.001 ** ^c^	-0.021	0.220	0.015	0.307	0.130	0.001 **	0.046	0.005 *
**Vf** ^b^	-0.086	0.325	0.168	0.055	-0.006	0.854	0.021	0.577	-0.018	0.670	-0.017	0.684	0.123	0.022 *	0.038	0.442
**Hb**	0.034	0.760	-0.082	0.384	-0.008	0.869	-0.038	0.433	-0.052	0.151	-0.060	0.129	0.069	0.143	-0.001	0.982
**Af**	-0.005	0.965	-0.032	0.709	0.050	0.168	0.053	0.150	0.004	0.921	0.069	0.103	0.199	0.001 **	0.127	0.012 *
**Is**	-0.106	0.217	-0.073	0.300	-0.074	0.395	-0.101	0.112	0.021	0.683	-0.011	0.772	0.064	0.404	-0.012	0.855
**Gb**	0.166	0.001 **	0.197	0.001 **	0.029	0.351	0.013	0.674	-0.007	0.822	-0.018	0.572	0.085	0.021 *	0.035	0.350
**Ba**	0.021	0.610	0.055	0.232	0.014	0.664	-0.058	0.080	-0.028	0.474	0.057	0.205	0.091	0.011 *	0.047	0.226

^a^All seven sites together.

^b^The abbreviations and the corresponding explanations of body sites are given in Table 2.

^c^*: *P*<0.05,

**: *P*<0.001.

We used Random Forest supervised learning models to determine the extent to which skin-associated microbial communities could be used to predict the age, gender, place of residence, or skin surface environment of the participant from whom a sample was taken from. Models were unsuccessful at determining the gender or body site associated with each sample. By contrast, the models performed approximately 4 times better than expected by chance at determining whether a sample was taken from an adolescent or an adult, and performed about 4.7 times better than expected by chance when determining which environment (urban vs rural) the sample’s participant resided. In both cases, model error increased greatly when trained on genus- or family-level taxonomic assignments instead of on OTUs.

The 3 samples taken for each body site across 3 non-consecutive days (Monday-Wednesday-Friday) had stable species richness (Table 2, S3 Fig), with very similar inter- and intra-sample weighted UniFrac distances (Fig 3E, ANOSIM *R* = 0, *P* > 0.05 with weighted UniFrac). Procrustes analysis (S6 Table) demonstrated that the bacterial community structure variation between the 1-day intervals was much higher on sites Hb and Vf; but extremely low on sites Ba and Na.

## Discussion

Our study confirmed that bacterial community structure is significantly different between body sites [4, 22, 23], and that the skin microenvironment type (sebaceous, moist and dry) were the most important factors influencing community structure [4]. Multiple host factors, including age, gender and place of residence, contributed to the variability in microbial distribution. We detected 4 physical skin parameters across 6 body sites (all except Na) that correlated with changes in the relative abundance of specific bacterial taxa. Although the correlation between *Propionibacterium* and sebum content has already reported [24], we found that the relative abundance of *Propionibacterium* also correlated with skin moisture. Age influences the skin microenvironment and thus the bacterial communities that reside there [25, 26]. The change in skin-associated bacterial community structure and composition during the first year of life shows that age can significantly influence diversity estimates [27, 28]. In the current study, the diversity of the skin microbiota in adults was significantly greater than in adolescents or the elderly. Skin bacterial communities adapt through time, utilizing carbohydrates, proteins, lipids, and minerals present on the skin surface [29]. The ability of the skin to hold moisture and its sebum production capacities are affected by aging as well as by gender [30]. Lipophilic bacteria such as *Propionibacterium*, start to increase in abundance during adolescence and peak during the third decade of life, which parallels sebum levels [25]. *Propionibacterium* prefer an environment with higher moisture and sebum. Males, who have greater sebum secretion that remains stable with ageing [30], had a greater relative abundance of *Propionibacterium* bacteria, which increased with age.

Physiological differences between male and female skin environments, such as hormone metabolism, perspiration rate and skin surface pH, can also account for gender differences [31, 32]. Although previous studies have only shown a significant difference in alpha diversity between men and women on one body site (palm) [6], the current study found this difference on virtually all body sites, especially Gb. Although any possible explanation of the gender differences associated with Gb would be supposition; it is possible that assumed differences in facial cosmetic application within this population could play a role.

Urban and rural populations had significantly different community composition, with greater intragroup variation among rural dwellers. Although urban versus rural environments are often significantly different, the lifestyle of the residence can also vary. The rural adults and elderly included in this study were all agricultural field-workers, whereas most urban participants had indoor occupations. These differences may alter skin conditions hence the bacteria that reside there [1, 33], but the sources from which a skin microbiome may populate would also be different. Indoor microbiomes are predominantly human-derived [11], whereas outdoor workers will be subject to soil, aquatic and host-associated microbial sources that could alter their skin microbiome composition.

In this study, the sequence reads per sample was relatively low (the average was 458 sequence reads per sample), however, we were able to get 3 replicate samples within 1-day intervals, and this read depth does cover the most abundant taxa on the skin surface [11]. Similar read depths had no influence on taxonomic correlations, with robust relationships observed across 100–400 reads per sample for skin studies [8]. The advantages of having large numbers of samples at shallow coverage clearly outweigh having a small number of samples at greater coverage for many datasets [34, 35]. The reproducibility of sequencing results were constant with previous reports pointing to the relative stability of the skin microbiome over time in the same individual [4, 5]. In addition, Procrustes analysis has demonstrated that partially occluded sites such as Ba and Na are more stable than other body sites [1], which supports our observation that Ba and Na had the lowest intragroup variation.

There is intensive interest in the variation of the human microbiome in relation to health and disease [36]. Dysbiosis of the microbial-host relationship, even in the absence of an invading pathogenic organism, may be influential in diseases such as primary immunodeficiency and atopic dermatitis [37]. The level of variability within a semi-contiguous human population, within the same geographic region, suggests that characterizing the microbial community structure of these different cohorts will be necessary if we are to use skin microbiome information for diagnosis and treatment.

Our study confirmed that bacterial community structure is significantly different between different body sites, and that the skin microenvironment types were the most important factors. Multiple host factors, including age, gender and place of residence, also contributed to the variability of the microbial distribution. Urban and rural populations showed significantly different community compositions, potentially due to the different skin condition as a result of work-type (office versus field) and the significant difference in microbial sources from which to populate their microbiome. Furthermore, we detected 4 physical skin parameters (sebum, TEWL, moisture and pH) across the body sites that correlated with changes in the relative abundance of specific bacterial taxa.

## Supporting Information

S1 Fig The distribution of OTUs. The horizontal axle represents the 13,004 qualified OTUs. The singletons in sequenced data were removed and the remained singletons were caused by the removal of samples with <200 reads. The vertical axle is the number of sequences in each OTU. To fit graphing, the reads numbers were transformed to logarithm.
(PDF)

S2 Fig UPGMA clustering of weighted (A) and unweighted (B) UniFrac distance of different groups of samples. The branch color means different sites, red branch: Na, yellow branch: Gb and Ba, blue branch: Is and Af, green branch: Hb and Vf. The sample names from different sites with same gender, age and place of residences were showed with same color.
(PDF)

S3 Fig Rarefaction of different alpha diversity indices (Chao1, PD and Shannon) by site, age, gender, place of residence and replication. (PDF)

S1 Table Analysis of similarity (ANOSIM) results for groups divided by different factors (single factors or multi-factors) used UniFrac distance. (XLSX)

S2 Table Comparison of relative abundance of bacterial genera between different groups with ANOVA (only showed *P*<0.05). All *P* values were corrected by the Bonferroni procedure for multiple comparisons.
(XLSX)

S3 Table The 20 major taxa abundances of 84 different group samples. The table matched to Fig 4.
(XLSX)

S4 Table Diversity indices of different group samples and comparison of different groups with t-test (only showed *P*<0.05). (XLSX)

S5 Table Analysis of Pearson-correlation for skin physical parameters (sebum, TEWL, moisture and pH) with contents of different genera. All *P* values were corrected by the Bonferroni procedure for multiple comparisons.
(XLSX)

S6 Table Procrustes analysis compared coordinate matrices of UniFrac distances for 3 replications on different sites. (XLSX)
