# Supplementary figures and images for: The Influence of Age and Gender on Skin-Associated Microbial Communities in Urban and Rural Human Populations
Source: PLoS One. 2015 Oct 28;10(10):e0141842. doi: 10.1371/journal.pone.0141842 (PMC4624872; doi:10.1371/journal.pone.0141842)

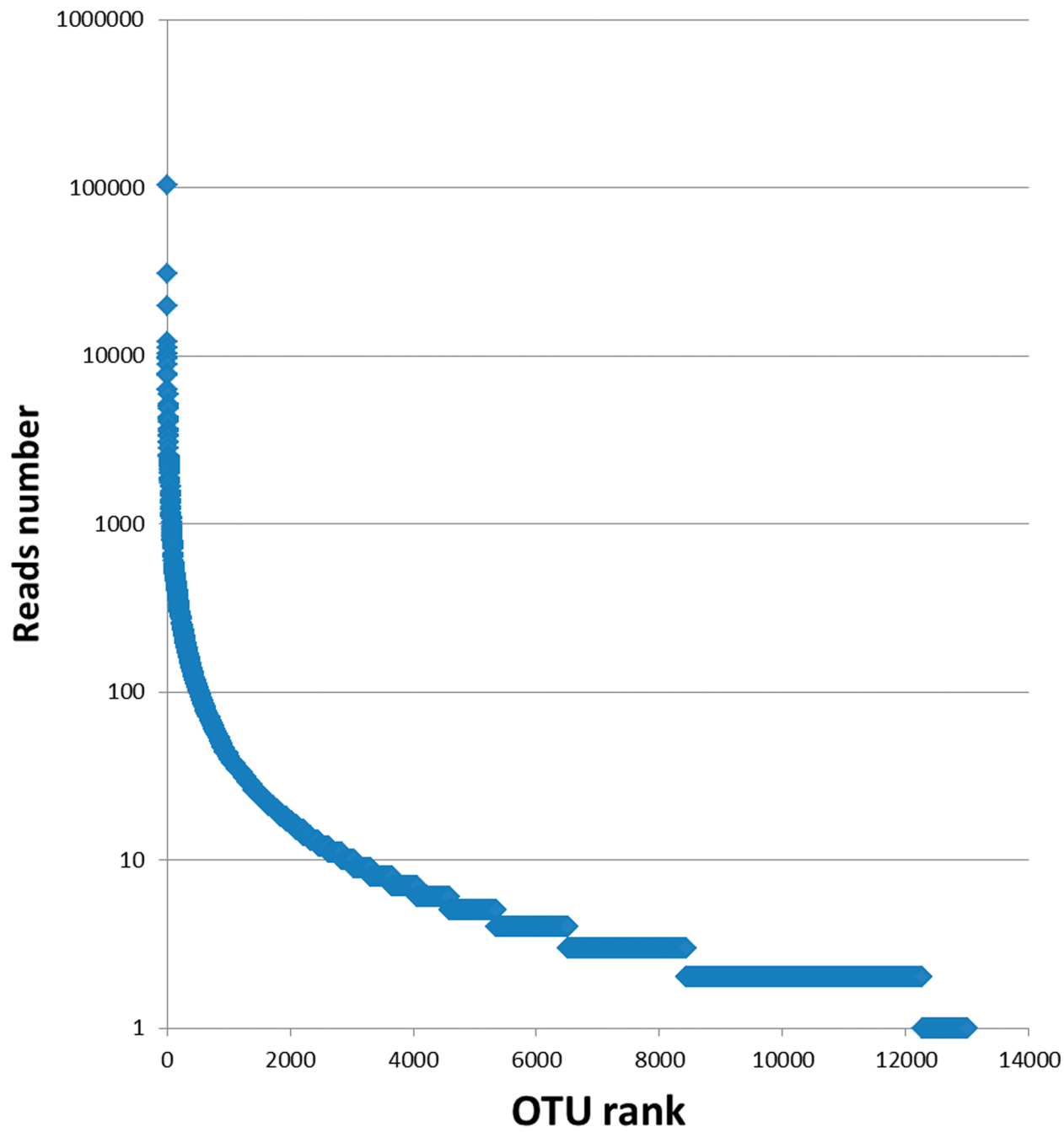

Supplement: S1 Fig — The horizontal axle represents the 13,004 qualified OTUs. The singletons in sequenced data were removed and the remained singletons were caused by the removal of samples with <200 reads. The vertical axle is the number of sequences in each OTU. To fit graphing, the reads numbers were transformed to logarithm. (PDF) [file pone.0141842.s001.pdf]

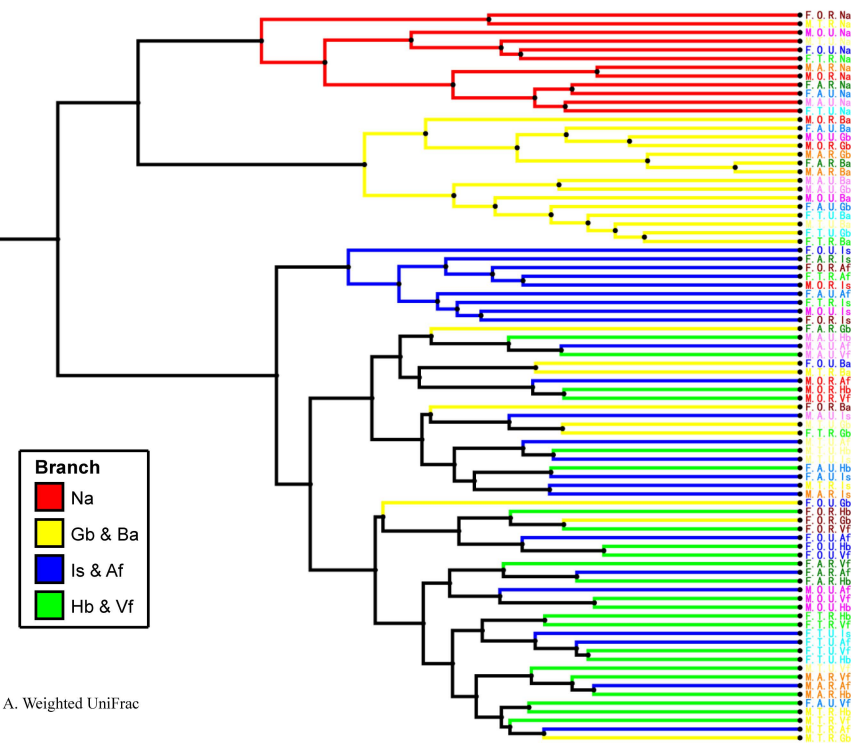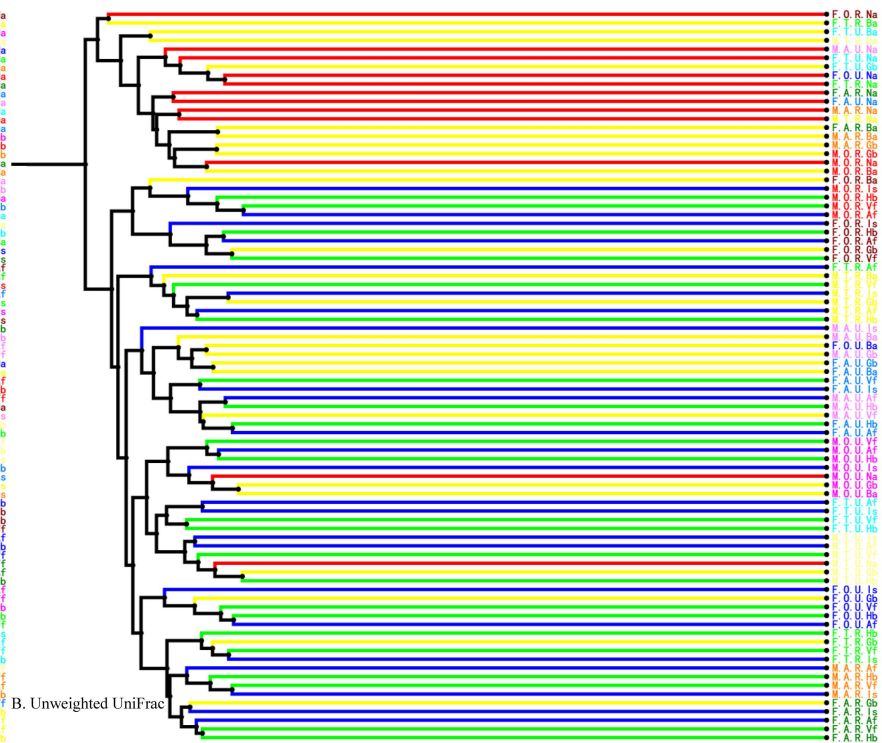

Supplement: S2 Fig — The branch color means different sites, red branch: Na, yellow branch: Gb and Ba, blue branch: Is and Af, green branch: Hb and Vf. The sample names from different sites with same gender, age and place of residences were showed with same color. (PDF) [file pone.0141842.s002.pdf]

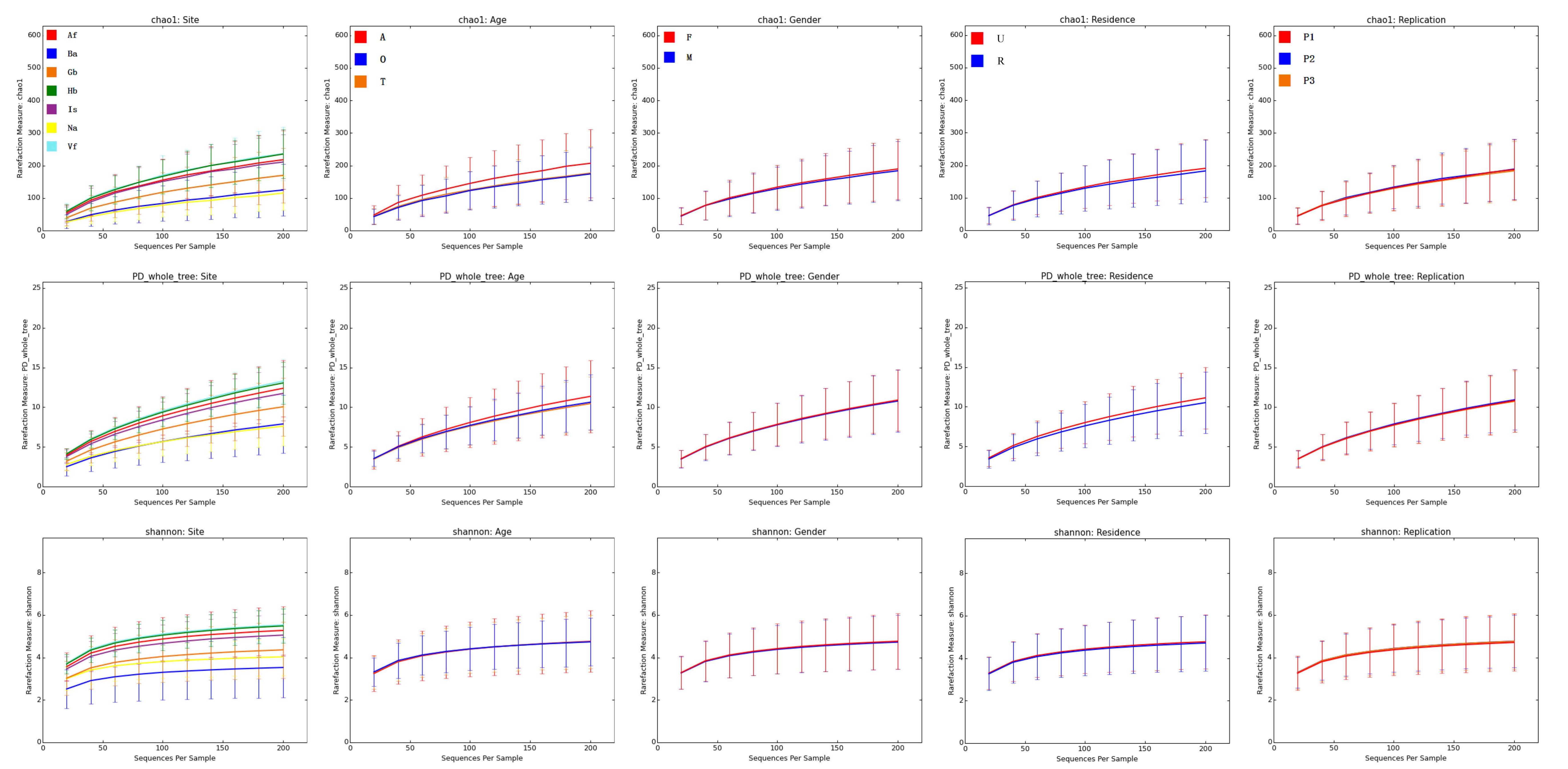

Supplement: S3 Fig — (PDF) [file pone.0141842.s003.pdf]
